# Investigating lexical knowledge and lexical processing in order to explore their role in second language utterance fluency

**DOI:** 10.1007/s10339-026-01396-8

**Published:** 2026-09-10

**Authors:** Nel De Jong, Mary Lou Vercellotti

**Affiliations:** 1 Vrije Universiteit Amsterdam, Amsterdam, Netherlands; 2 Department of English, Ball State University, Muncie, USA

**Keywords:** L2 speech, Cognitive fluency, Pausing, Syllable duration, Picture naming task, Productive tasks

## Abstract

Research has found that lexical knowledge substantially influences second language (L2) fluency. However, researchers investigating the role of L2 lexical knowledge on speakers’ fluency have often measured lexical knowledge with written recognition tasks, which creates a mismatch between the assessment modality and the oral, productive skill. Furthermore, although some studies have considered the role of lexical processing (e.g., lexical retrieval and articulation) when researching utterance fluency, results have been mixed. The current study adds to this scholarship by using mainly productive tasks to investigate possible relationships among measures of L2 lexical knowledge and processing and to explore their role in L2 utterance fluency. High-intermediate language learners (*n* = 30) in an intensive English program were assessed on their lexical knowledge (i.e., form recall, meaning recognition and sentence production), oral processing (i.e., lexical retrieval speed), and articulation (i.e., preparation time and duration). Utterance fluency was measured with pause length, number of pauses, and syllable duration during a monologic topic-based speech. Correlational analysis revealed a negative correlation between form recall and lexical retrieval time, meaning that L2 learners who could recall more words retrieved them faster. Regression analyses revealed that lexical meaning scores predicted pause length, articulation preparation time predicted mean number of pauses, and lexical retrieval predicted syllable duration. The results suggest that the lexical knowledge, lexical retrieval speed, and the phonological/phonetic system (i.e., articulation preparation) are clearly and differentially involved in utterance fluency in high-intermediate speakers.

## Introduction

While the term *fluency* is often used colloquially as a synonym of proficiency, fluency is a sub-construct of L2 proficiency in second language (L2) research, along with complexity, accuracy, and lexis (Norris and Ortega 2009; Vercellotti 2017), which can assess language proficiency. Perceived fluency is a subjective judgement of a speaker’s fluency whereas utterance fluency is measured objectively with pausing phenomena and by the speed with which words are articulated (Segalowitz 2016).

Certainly, accessing lexical items quickly is important for reading, writing, listening, and speaking (e.g., Schmitt 2014), but L2 research has suggested that lexical knowledge is particularly relevant to speaking skills (e.g., De Jong et al. 2012; De Jong and Vercellotti 2016; Hilton 2008; Koizumi and In’nami 2013; Suzuki and Kormos 2023; Takizawa 2024; Uchihara and Saito 2019). Struggling to find a word lowers utterance fluency because fluency depends on the appropriate words being known, retrieved sufficiently quickly, and articulated at a reasonable rate. Indeed, lexis plays a central role in Levelt’s (1999) speech production model; and lexical processing, the speed and efficiency of the processes needed for speech, is a key component of Segalowitz’s (2016) framework of L2 fluency. Accordingly, we may explain L2 utterance fluency by identifying the processes responsible for disfluencies in speech (Segalowitz 2016).

Unfortunately, there are several issues which complicate our understanding of the research about lexical knowledge, lexical processing, and their role in L2 utterance fluency. Firstly, the mental lexicon is complex, particularly in L2 language learners, and there is no consensus on how to measure lexical knowledge (Schmitt 2014). Secondly, although Segalowitz’s (2010) model of L2 fluency highlights the importance of lexical processing, few studies of lexical knowledge also capture lexical processing information. As a result, little is known about the independence of measures of lexical knowledge and lexical processing. Thirdly, there is relatively little research investigating the role of lexical processing and lexical knowledge on L2 utterance fluency even though lexical retrieval, the speed and automaticity with which words are accessed (Hulstijn 2015; Schmitt 2010), is crucial to utterance fluency. Furthermore, the results from existing research (e.g. Clenton et al. 2020; De Jong et al. 2012; Kahng 2020) about the role of lexical knowledge and processing on utterance fluency has been mixed. For example, Uchihara and Clenton (2022) found that vocabulary knowledge did not correlate with fluency during a speaking task whereas Uchihara and Saito (2019) reported a moderate correlation between vocabulary knowledge and fluency. Overall, the influence of lexical knowledge and processing on L2 utterance fluency has not yet been thoroughly investigated (Miralpeix and Muñoz 2018; Uchihara and Saito 2019). This exploratory study addresses this empirical gap by investigating L2 lexical knowledge and lexical processing and then explores how they might influence utterance fluency of L2 speakers of English from various language backgrounds.

## Literature review

### L2 lexical processing and lexical knowledge

This research study focuses on two aspects of L2 cognition: L2 lexical processing and lexical knowledge. In the following subsections, each is discussed, as well as relevant methodological issues about researching lexical processing and lexical knowledge.

#### Lexical processing speed

Lexical processing includes the ability to retrieve words and the time needed to articulate the words. First, although Segalowitz (2010) identified speed of lexical retrieval as a component of L2 cognitive processing, overall, relatively few studies have measured lexical processing when studying L2 lexical knowledge. Theoretically, lexical processing speed and lexical knowledge may be related because if an L2 learner knows many words, lexical processing might be slowed as the learner attempts to select the appropriate word. Llompart (2021), however, found that advanced German first language (L1), English L2 learners with larger vocabularies made faster lexical decisions. It is not clear whether this relationship is generalizable to all L2 learners or whether it is limited to learners of related languages (e.g., German and English) or to advanced L2 learners.

Secondly, according to Levelt (1999), the final step in speech production is the articulation of words. Articulation includes both the time the speaker needs to start articulating a word after retrieving it (articulation preparation time) and the time the speaker needs to completely articulate a word (articulation duration). To measure lexical retrieval and articulation preparation time, participants are presented with a stimulus and are asked to name the item as quickly as possible either immediately or after a short delay. Under the delayed naming condition, lexical retrieval can be completed before the response time measurement starts, so response times tap only late processes (i.e., articulation preparation). In contrast, under the immediate naming condition, response times include both early processes and late processes (e.g., Broos et al. 2021). Consequently, lexical retrieval speed will be correlated with articulation preparation time because saying a word in the immediate naming condition includes both the time it takes to retrieve the word and the preparation time it takes to begin to articulate the word. Articulation duration (sometimes called response duration) is measured during the delayed picture naming task (e.g., De Jong et al. 2012; Kahng 2020).

It should be noted that articulation speed measures could be related to features of the language itself (e.g., Broos et al. 2021), such as the number of phonemes per syllable (De Jong et al. 2015), or to individual speaking style (e.g., Kahng 2020), which suggests that the articulation measures (i.e., articulation preparation time, articulation duration) may capture more than the processes that influence utterance fluency. More research is needed to investigate the relationships among these lexical processing skills (i.e., lexical retrieval, articulation preparation time, and articulation speed) in learners who have not yet reached advanced proficiency and in English L2 learners with various language backgrounds, particularly given that previous research (i.e., Broos et al. 2021; Llompart 2021) studied advanced bilinguals with L1s related to English.

#### Lexical knowledge

Although lexical knowledge begins with making the connection between the word’s form (e.g., pronunciation, spelling) and its meaning, or more precisely, one of its meaning senses (Stoeckel et al. 2021), lexical knowledge is multifaceted (Nation 2001; Schmitt 2014). Knowledge of lexis builds incrementally, and some types of lexical knowledge are likely learned before others (Chui 2006; González-Fernández and Schmitt 2020; Schmitt 2010). While various aspects of vocabulary knowledge are important for pedagogical purposes, lexicon size is often measured when conducting research (Read and Dang 2025). The size of a learner’s lexicon is the number of words known, or known to a particular level (Schmitt 2014).

Some measures of lexical knowledge have been found to correlate, suggesting that they develop in parallel (Laufer et al. 2004) or reflect the same construct (Koizumi and In’nami 2013). The strength of the correlation, however, varies across testing formats (González-Fernández and Schmitt 2020). For instance, research has shown that lexicon size is typically larger receptively than productively (e.g., Laufer and Goldstein 2004). In other words, L2 learners understand more words than they produce in their speech or writing. Receptive knowledge may only require a connection between the word’s form and its meaning (Schmitt 2014). When L2 learners have only partial knowledge of a word’s meaning, they may be able to recognize it but may not be able to recall the word or to use it appropriately in a sentence. As a result, receptive tests might not reflect their ability to use this lexical knowledge in productive language communication (Schmitt 2014). And, this lag would be expected to be larger in instructed language learners because instruction introduces many vocabulary words, which can be memorized. Thus, measuring both productive lexical knowledge and receptive lexical knowledge is important for understanding lexical knowledge (González-Fernández and Schmitt 2020).

Moreover, different lexical knowledge tasks can reveal the strength of the form-meaning connection. For instance, recalling a word’s form is more difficult than understanding a word once the word form has been given (Schmitt 2014) and producing a sentence for a given word is more difficult than recalling its meaning (e.g., Chui 2006). In other words, passive form recognition is easier than active form recall and meaning recognition is easier than producing a sentence that demonstrates word meaning (Naismith 2022). Measuring lexical knowledge with passive, receptive tasks, however, is easier, and few research studies have measured lexical knowledge with productive tasks (Enayat and Derakhshan 2021).

While some studies investigating lexical knowledge and utterance fluency (e.g., Uchihara and Clenton 2022; Uchihara et al. 2022; Uchihara and Saito 2019) measured productive lexical knowledge, they used a word association task (Lex30), which asks participants to give multiple responses to each lexical cue. A word association task is considered a measure of depth of vocabulary knowledge (Read and Dang 2025). The scores of such tasks, therefore, conflate separate aspects of lexical knowledge because it asks for multiple responses (lexicon size) to show word association knowledge (lexical depth). More importantly, a word association task does not approximate the linguistic task required to retrieve and articulate lexical items in speech for communication purposes. Accordingly, more research with other productive measures of lexical knowledge is needed to better understand the connections between lexical knowledge and utterance fluency.

In addition to the use of receptive tests, many previous studies (e.g., Chui 2006; De Jong et al. 2012, 2015; Enayat and Derakhshan 2021; Gao and Sun 2025; González-Fernández and Schmitt 2020; Kahng 2020; Koizumi and In’nami 2013; Suzuki and Kormos 2023; Takizawa and Suzuki 2026) have used written tests to measure vocabulary size or the cues for the lexical knowledge test were given in written form (e.g., Uchihara and Clenton 2022). The use of written language for vocabulary tests conflates lexical knowledge with decoding and literacy. Orthographic lexical knowledge for L2 language learners, of course, also depends on the writing system used by the learner (Schmitt 2014), so the difficulty of the task can differ across L2 learners from different L1 backgrounds. For these reasons, measuring lexical knowledge using solely written tests can be problematic, despite their convenience. In fact, Uchihara and Clenton (2022) concluded that oral vocabulary tests should be used if the goal is to investigate how lexical knowledge affects utterance fluency.

In sum, L2 lexical processing includes lexical retrieval speed, articulation preparation time, and articulation duration. The relationships among measures of L2 lexical processing and lexical knowledge across learners of English from various L1 backgrounds is unknown. And, although lexical knowledge has been more commonly researched, there are limitations of measuring lexical knowledge with receptive tasks or only written tests. Productive tests, such as active recall and using the word in a sentence, better approximate how lexical knowledge is used in communication. More research is needed to understand the connections between lexical processing and lexical knowledge in order to explain L2 utterance fluency of instructed English language learners across L1s.

### Utterance fluency

L2 utterance fluency reflects the characteristics of overt speech. Utterance fluency includes breakdown fluency and speed fluency (e.g., Bosker et al. 2013).^1^ Breakdowns in fluency, or interruptions in the flow of speech, are most evident as silence and non-lexical filler words (e.g., “uh”). Empirical research has found that silent and filled pauses are unrelated (e.g., De Jong et al. 2012, 2015) or negatively correlated (e.g., Bosker et al. 2013) in speech, which indicates that individual speakers tend to use either filled pauses or silent pauses when experiencing fluency breakdowns (e.g., Bosker et al. 2013; De Jong et al. 2012).

Speed fluency is independent of breakdown fluency and is often measured as articulation rate or mean syllable duration (Takizawa 2024). Articulation rate is the number of syllables divided by speaking time (e.g., Kallio et al. 2022). Articulation rate in L2 English learners has been found to increase over time (De Jong and Perfetti 2011) and to be independent of prompt (De Jong and Vercellotti 2016), which indicates that articulation rate reflects language proficiency. In fact, De Jong et al. (2012) concluded that articulation rate or its inverse, mean syllable duration, may be the fluency measure that aligns the most with L2 proficiency. Of note, mean syllable duration is a measure of disfluency, where a higher mean reflects lower fluency (De Jong et al. 2012; Kahng 2020). The field has used other measures of utterance fluency, such as mean length of run and speech rate, but these measures are composite measures because their calculation includes pausing information (Yan et al. 2025). Instead, it is recommended that researchers use multiple measures of fluency to capture the different sub-constructs (Yan et al. 2025). The disfluency measures of the mean number of pauses, mean pause length, and mean syllable duration are pure (unconfounded) measures and complementary measures, in that they capture different aspects of utterance fluency.

### Connecting lexical processing and lexical knowledge to utterance fluency

Levelt’s (1999) model provides a framework to connect different types of lexical knowledge and processing to fluency during speech production (i.e., utterance fluency). In Levelt’s (1999) model, speech production starts with conceptualization, followed by grammatical encoding, in which lemmas and their syntactic frames are activated. Next, morpho-phonological encoding, phonetic encoding, and articulation lead to the production of overt speech. Segalowitz (2010) has argued that the stages in Levelt’s (1999) production model are fluency vulnerability points, as difficulties may arise during conceptualization, grammatical encoding, or articulation. Intuitively, an L2 speaker may pause (silently or with a filler) while thinking about the content of the message, when encoding the message into the target language, and/or when articulating the message. Disfluencies in any of these cognitive processes can result in disfluency in speech (Segalowitz 2016). Speakers may even lengthen syllables while planning upcoming language during articulation (Duez 1982; Hilton 2014), resulting in a longer mean syllable duration. In other words, linguistic resources (e.g., lexical knowledge) and linguistic processing (e.g., lexical retrieval speed, articulation time) can both affect utterance fluency (Suzuki and Kormos 2023). Therefore, L2 research can investigate whether these cognitive vulnerability points empirically connected to specific measures of utterance fluency. For instance, disruption during conceptualization has been found with pause-related measures, but not with articulation rate (e.g., De Jong and Vercellotti 2016). And, Gao and Sun (2025) concluded that lexical retrieval speed, which is an encoding vulnerability point, impacts both pause rate and pause duration.

Of course, lexical knowledge is crucial for language production. Specifically, lexicon size has been found to be related to success in spoken production (e.g., Koizumi and In’nami 2013; Miralpeix and Muñoz 2018; Takizawa and Suzuki 2026; Vercellotti 2017). Lexicon size may be especially consequential to utterance fluency because speakers who can actively recall a larger number of lemmas may be more likely to find an appropriate lemma in time for processing to proceed with shorter interruptions (shorter pauses) or without interruption, resulting in fewer pauses and longer stretches of language. Alternatively, knowing more words may increase pause length as the speaker must select the most appropriate word from several options. Hilton (2008) found that lexical searches often resulted in very disfluent speech, with several pauses, including very long pauses. Although lexical knowledge, and specifically productive lexical knowledge, is critical to L2 utterance fluency, it is not yet clear whether lexical knowledge measured as recall of form or lexical meaning better facilitates lemma selection, which then increases fluency in speech.

In addition to lexical knowledge, research has found that L2 utterance fluency is affected by lexical retrieval speed in English L2 speech (De Jong et al. 2012; Gao and Sun 2025) and Spanish L2 speech (Segalowitz and Freed 2004). Beyond lexical retrieval, Broos et al. (2021) concluded that for advanced bilinguals, articulation, specifically phonetic planning, is the likeliest source of slower articulation. Thus, it is possible that the time required for articulation could explain pausing in utterance fluency in L2 speech.

Recent studies have attempted to connect lexical knowledge and lexical processing, including lexical retrieval speed, with specific measures of English L2 utterance fluency. Kahng (2020) investigated possible relationships between lexical knowledge and processing and utterance fluency in intermediate learners of English while Clenton et al. (2020) investigated them in pre-intermediate L2 learners of English. Gao and Sun (2025) explored the relationships with learners of English across proficiency levels, from lower intermediate to advanced. First, the results about pausing are mixed. Both Kahng (2020) and Clenton et al. (2020) concluded that greater lexical knowledge correlated with higher fluency as measured as the number of silent pauses. In contrast, in Gao and Sun’s (2025) study, their pure measures of lexical knowledge did not predict utterance fluency measured as pause rate. Gao and Sun (2025) reported that lexical retrieval speed predicted silent pause length whereas neither Clenton et al. (2020) nor Kahng (2020) found any correlation between the lexical knowledge or processing measures and mean silent pause length.

Second, lexical retrieval speed was inconsistently related to utterance fluency. Kahng (2020) reported a positive correlation between lexical retrieval speed and the number of mid-clause (silent and filled) pauses, but Clenton et al. (2020) found no correlation between lexical retrieval speed and pausing. Likewise, even though Kahng (2020) and Clenton et al. (2020) both reported a correlation between lexical retrieval speed and syllable duration, Kahng reported a positive relationship but Clenton et al. reported a negative relationship. In contrast, Gao and Sun (2025) reported that L2 lexical retrieval speed was not relevant to L2 articulation rate (the inverse of syllable duration).

Finally, Clenton et al. (2020) found that articulation preparation time was positively correlated with the number of silent pauses (of at least 350 ms), which means that speakers who take longer to begin the articulation of words produce more pauses. They also found that articulation preparation time was negatively correlated with mean syllable duration, which suggests that learners with faster articulation preparation time articulated syllables more slowly; Clenton et al. (2020) suggested that more research is needed to understand this counter-intuitive finding. Overall, the results from these studies do not present a clear picture of the role of L2 lexical knowledge and processing in L2 utterance fluency.

Furthermore, several of the studies that investigated the role of lexical knowledge and lexical processing on utterance fluency were limited to L2 learners of English from a single language background (e.g., Broos et al. 2021; Clenton et al. 2020; Gao and Sun 2025; Kahng 2020; Koizumi and In’nami 2013; Suzuki and Kormos 2023), and some studies (e.g., Gao and Sun 2025; Takizawa 2024) used a measure of lexical knowledge chosen for that shared L1-L2 population. Given the limitations of conclusions drawn from studies with homogenous participants, Enayat and Derakhshan (2021) suggested exploring lexical knowledge and speaking proficiency of participants from different cultural and linguistic backgrounds. A study with L2 learners of English with various L1 backgrounds offers additional insight on which patterns are generalizable across English L2 learners.

Overall, more research is warranted to understand L2 lexical knowledge and processing, particularly using productive and oral measures of lexical knowledge and to explore which specific lexical knowledge and lexical processing measures predict distinct aspects of utterance fluency for learners from different language backgrounds. Our research questions (RQ) are:

- RQ1: For high-intermediate L2 English learners from various language backgrounds, do the measures of lexical processing (i.e., lexical retrieval, articulation preparation time, articulation duration) and lexical knowledge (i.e., lexical recall of form, lexical meaning) correlate?
- RQ2: Which measures of lexical processing or lexical knowledge, if any, predict utterance fluency (i.e., mean pause length, number of pauses, mean syllable duration) in the speech of high-intermediate L2 English learners with mixed L1s during a topic-based speaking task?

## Methodology

This study was conducted within an intensive English program (IEP) at a large university in the U.S. The classroom context limited the number of possible participants as well as the number and length of the research tests. This research design, however, enabled data collection from the target population of instructed learners while avoiding issues related to self-selection bias (e.g., motivation, willingness to communicate) and thus, reflects a broader population of English L2 learners.

### Participants

Participants were all students enrolled in the Speaking course at the high-intermediate level, and most students were simultaneously enrolled in reading, writing, listening and grammar courses. This IEP used an in-house placement test upon enrollment. Students placed at the high-intermediate level were approximately B1 of the Common European Framework. Thirty participants (mean age 27, range 18 to 44; 13 male, 16 female; one participant chose not to disclose personal information) attended both data collection sessions. First languages included Arabic (3), Chinese (6), French (1), Italian (2), Japanese (4), Korean (5), Russian (1), Slovak (2), Spanish (2), and Turkish (3).

### Procedure

Data collection occurred as a class activity in all sections of the course. Before beginning the data collection, each participant signed a consent form for their data to be used for this educational research. The participants completed the tasks simultaneously and individually in the university’s language learning computer lab during their section’s class meeting time. The participants completed the lexical knowledge test followed by the lexical meaning test during one class meeting. The participants completed the monologues one to three days later, as part of the existing speaking class curriculum. The researchers were simply given access to the recordings of the speeches for the present study.

### Materials

We assessed the size of participants’ L2 lexical knowledge^2^ using an oral form recall task with picture prompts. Although picture-naming is the standard way to measure lexical retrieval speed, this task is also a valid measure of vocabulary knowledge because form recall is an acknowledged measure of vocabulary size. Another common form recall task, L1/L2 translation, was not chosen as we wanted to avoid reading skills for the vocabulary size test. Picture prompts remove any orthography advantage or disadvantage, given that the participants in this study came from various language backgrounds. Participants named 18 target words in the immediate picture-naming task and 18 different words in the delayed picture-naming task. The 36 target words, all nouns because of the visual format, were selected from the 4000 most frequent lemmas in the general frequency list in the Corpus of Contemporary American English (COCA; Davies 2008).^3^ Although high-intermediate learners may recognize most words at this frequency, their ability to recall them would be less likely (González-Fernández and Schmitt 2020); therefore, we expected that these words would distinguish learners within this instructional level. All pictures had been previously normed (e.g., Snodgrass and Yuditsky 1996; Székely et al. 2003).

For the picture naming task, each trial started with a fixation cross in the middle of the screen for one second. In the immediate naming condition, a picture appeared with a red circle to indicate that the participant should name the picture; after seven seconds, the audio recording stopped, and the circle turned gray. In the delayed picture-naming condition, the picture was shown with a gray circle for three seconds, which turned red when a beep prompted the participant to name the picture; after four seconds, the audio recording stopped and the circle turned gray. Audio recording took place during the entire seven seconds. Finally, the correct answer appeared below the picture, as required by the cooperating IEP, so that participation in the research would be pedagogically beneficial. After three seconds, the next trial began.

We assessed the participants’ L2 lexical meaning knowledge with a receptive test of definitional knowledge and productive sentential knowledge (i.e., ability to use the word meaningfully in a sentence), with an adaptation of the Vocabulary Knowledge Scale (Wesche and Paribakht 1996). We chose a task that asked participants to use the lexical item in context because it approximates the cognitive processes required to use lexical knowledge during communication. Target words for the lexical meaning test were nine nouns and nine verbs, with a mean COCA lemma frequency of 11,620 (*SD* = 12,672, minimum 80, maximum 43,003). The answer options, which averaged 3.8 words, were not exact definitions but somewhat broad descriptions of the meaning of the target word. Distracter definitions were selected and adapted from the Vocabulary Levels Test, Test B (Nation 2001) and were definitions of words with similar frequencies to the target word.^4^ The words, frequencies, and answer options are available in IRIS.

The lexical meaning test first presented a target word and multiple-choice options to report how familiar the participants were with the word. Whenever participants answered that they were familiar with the target word’s meaning, three randomly ordered definitions appeared. If they indicated that they could use the word in a sentence, a textbox also became active so that they could demonstrate sentential knowledge.^5^ Whenever a participant indicated that they knew a word but neglected to select a definition and/or write a sentence, missing elements were highlighted. The test allowed participants to change their responses until they clicked the Continue button and a progress bar at the bottom of the screen indicated the one-minute time limit per word. The picture naming and lexical meaning tests were custom created for this study and run with Flash Player on Dell computers in the university computer lab.

The minute monologic speeches were part of a computer-aided graded activity in the IEP’s curriculum. (See McCormick and Vercellotti 2013 for a description of the activity.) Three topics were discussed in class in the week before the speech, but students were not informed which topic would be presented for the classroom assignment. The participants had one minute for preparation without the use of aids or note-taking, which were the IEP’s instructions for this course activity. During the recording of the two-minute monologue, a clock on screen showed the time remaining. The analyzed speeches were elicited with the prompt “Describe an event in your country’s history. Do you think this event was important? Why or why not? Give two or three reasons.”

### Data coding

To score lexical size, responses to the picture-naming task were coded as correct if a participant said the target word or a close synonym (e.g., *trousers* for *pants*), as judged by an expert speaker, following Székely et al. (2003). Responses during the delayed condition were considered correct whether they occurred before or after the response cue. The maximum score was 36.

To score lexical meaning knowledge, one point was awarded when a correct definition was chosen. For every produced sentence during the second step of the lexical meaning task, the sentence (*n* = 185) was reviewed individually by a trained English L1 research assistant and checked by the first author. A second point was awarded whenever the participant demonstrated knowledge of the target word’s meaning in a semantically appropriate sentence. Given that meaning is critical to lexical knowledge but grammaticality is not (Laufer et al. 2004), grammatical and spelling errors were not considered in the scoring. The score (max. 36), capturing both definitional knowledge and sentential knowledge, was a composite score of lexical meaning following Wesche and Paribakht (1996) (cf. Stewart et al. 2012).

The lexical processing and articulation measures were manually determined, using spectrograms. Lexical retrieval time was measured as the response latency in the immediate picture-naming condition, i.e., the time between the presentation of the picture and the onset of the response. Articulation preparation time was measured by the response latency in the delayed picture-naming condition, i.e., the time between the response cue (a beep) and the onset of the response. Incorrect responses and responses given before the response cue in the delayed condition were excluded from this measure. For the response duration (i.e., the time between the onset and the end of the response) only correct responses in the delayed condition were analyzed.

For the monologues, two trained research assistants transcribed all speeches using the PRAAT (Boersma 2001) function “To textgrid (silences)” set at 0.2 s to identify silent pauses, and these automatically identified boundaries were manually adjusted as necessary. This low pause threshold better captures automatized speech (e.g., Myles and Cordier 2017) and this threshold has been used in other L2 fluency research (e.g., De Jong and Vercellotti 2016; Hilton 2008; Vercellotti 2017). Non-verbal fillers such as “uh” over 0.2 s were transcribed and combined with the silent pauses because they both reflect cognitive disfluency (e.g., Myles and Cordier 2017). From the transcriptions, research assistants counted the syllables. To obtain a reliability measure, 36 randomly selected minutes of speech were recounted by the first author; the agreement between the two counts was 97%. Any discrepancy was usually only one syllable.

The mean pause (silent and filled) length was calculated for each participant. The upper limit for pauses was set to 2.5 standard deviations above the mean in a particular participant’s recording. Pauses longer than this (6.2% of all pauses) were replaced by the mean plus 2.5 standard deviations in the calculation of mean pause length. The number of pauses was normalized by computing the number of pauses (silent and filled) per 100 syllables in the speech, which gives a measure of pausing frequency that is independent from pause length and is not influenced by the amount of speech produced.

The mean syllable duration was calculated by dividing the amount of speech time (total time minus pause time) by the total number of syllables produced, following previous research (e.g., De Jong et al. 2012, 2015). Mean syllable duration is a preferable measure of speed fluency because the data tend to have a more normal distribution (De Jong et al. 2012).

These three utterance fluency measures (i.e., mean pause length, mean number of pauses, mean syllable duration) are complementary and unconfounded. Pure measures allow for better interpretation of the results (Suzuki et al. 2021) and can connect vulnerability points to specific utterance disfluencies. Table 1 summarizes the materials and measures.

**Table 1:** List of research measures

	Test	Description
*Lexical knowledge*		
Lexis size: recall	Picture naming tests	Productive, form recall, oral task
Meaning	Revised vocabulary knowledge scale	Recognition of meaning; sentence production
*Lexical processing speed*		
Retrieval speed	Immediate picture naming	Time between picture presentation and response onset
Articulation preparation time	Delayed picture naming	Time between response cue and response onset
Articulation duration	Delayed picture naming	Time between response onset and end
*Utterance fluency*		
Breakdown fluency	Two-minute monologues	Mean pause length
		Number of pauses/100 syllables
Speed fluency		Mean syllable duration

### Data analysis

Analyses of the lexical meaning test were done on 29 data sets because one participant did not complete this test. For the analyses of data from the picture naming task (lexis size, lexical retrieval, articulation preparation time, and articulation duration), three participants were excluded for not completing the tests. Three additional participants were excluded from the articulation preparation analysis because they did not respond at the cue as instructed in the delayed naming condition.

For lexicon size, reliability was acceptable (α = 0.78). The reliability of the lexical meaning test was good (α = 0.84). Since only correct responses were included, the reliability for the remaining tests could not be computed. Distribution of the data was normal for all measures (*p*s > 0.05 for the Kolmogorov-Smirnov test) in addition to visual inspection. We calculated bootstrapped 95% confidence intervals (CIs), using 1000 samples, simple resampling, and the bias-corrected and accelerated (BCa) method, which adjusts for any skewness in the bootstrapped data set and for any nonconstant variance (LaFlair et al. 2015).

To answer the first research question, we used Pearson correlation coefficients, with BCa 95% CIs, using 1,000 samples and simple resampling. To answer the second research question, we used forward stepwise multiple regression analyses with the five lexical knowledge and processing measures as the predictors and the three utterance fluency scores from the monologues as the dependent variables. Stepwise regression is appropriate for exploratory studies which attempt “to understand the relationship between variables” (Thayer 2002, p. 2); we chose this regression method, which enters predictors based on their unique contribution to prediction (Stevens 2002) because the literature does not suggest a particular order in which predictors should be entered into the model. Stepwise regression has been used in other recent studies (e.g., Gao and Sun 2025; Kahng 2020). Assumptions were met, including lack of collinearity, independence and normality of residuals, linearity, and homoscedasticity.

## Results

### Lexical knowledge, processing, and articulation

The participants’ lexical knowledge size scores from the lexis recall task (*M* = 29.6; *SD* = 3.6) ranged from 21 to 35 correct responses (max. 36), indicating that many words were known with some variation among participants. The participants’ lexical meaning scores ranged from 8 to 29 points (max. 36). The mean (18.8) suggested that participants on average could identify the meaning of the target words (a score of 1 per word) but were unable to produce a semantically appropriate sentence for them (a score of 2). The standard deviation (6.44) showed there was substantial variation among participants. A post-hoc review found that only participants who had above average lexical meaning scores demonstrated sentential knowledge of multiple words.

**Table 2:** Descriptive statistics and bootstrapped BCa 95% confidence intervals of lexical measures

Measure	*n*	Mean	*SD*	Range	BCa 95% CI for mean
Lexis recall (max. 36)	27	29.6	3.6	21–35	28.3, 30.9
Lexical meaning (max. 36)	29	18.8	6.44	8–29	16.6, 20.8
Lexical retrieval time (sec.)	27	1.74	0.43	1.13–2.86	1.59, 1.89
Articulation preparation (sec.)	24	0.83	0.27	0.34–1.32	0.72, 0.93
Articulation duration (sec.)	27	0.53	0.09	0.35–0.69	0.50, 0.56

Lexical retrieval time (*M* = 1.74; *SD* = 0.43) of known words ranged from 1.13 to 2.86 s, indicating substantial variation among these high-intermediate learners. Articulation preparation, the time it took the participants to begin articulating already retrieved words, (*M* = 0.83; *SD* = 0.27) also showed variation, whereas articulation duration (*M* = 0.53; *SD* = 0.09) showed little variation. Table 2 shows the descriptive statistics and bootstrapped confidence intervals of the lexical measures.

### Relationships between lexical knowledge and lexical processing

Since the lexical retrieval measure unavoidably includes articulation preparation (Kroll et al. 2002), the correlation between lexical retrieval speed and articulation preparation time was expected (*r* = .51, BCa 95% CI [0.21, 0.79]). As reported in Table 3, there was a significant correlation between lexis recall scores and lexical retrieval time (*r* = − .46, BCa 95% CI [-0.73, − 0.15]), which was fairly strong and negative. This correlation indicates that better lexical recall was related to shorter time lapses during lexical retrieval. In other words, L2 learners who could name more items named them more quickly.

**Table 3:** Pearson correlation coefficients for lexical measures

					BCa 95% confidence intervals			
	Meaning	Retrieval	Art. prep.	Art. dur.	Meaning	Retrieval	Art. prep.	Art. dur.
Recall	.03^c^	**− 0.46** ^a^	− .39^b^	.01^a^	− 0.33, 0.37	**− 0.73**,** − 0.15**	− 0.70, 0.10	− 0.38, 0.50
Meaning		− .22^c^	− .12^d^	− .11^c^		− 0.60, 0.30	− 0.61, 0.47	− 0.45, 0.30
Retrieval			**0.51** ^b^	.03^a^			**0.21**,** 0.79**	− 0.29, 0.36
Art. prep.				.33^b^				− 0.16, 0.69

Note. Art. prep. = articulation preparation; art. dur. = articulation duration

^a^ *n* = 27; ^b^ *n* = 24; ^c^ *n* = 26; ^d^ *n* = 23; significant correlations in bold

Notably, there was no evidence of a significant correlation for the two measures of lexical knowledge with a confidence interval ranging between weakly negative and weakly positive for these participants (*r* = .03, BCa 95% CI [–0.33, 0.37]). Likewise, these data did not show evidence for a significant correlation between lexical meaning and lexical retrieval time (*r* = − .22, BCa 95% CI [-0.60, 0.30]), with a confidence interval ranging from moderately negative to weakly positive.

### Utterance fluency

The participants’ utterance fluency was assessed via their performance during the two-minute classroom monologues. As shown in Table 4, the mean pause length was 1.19 s (*SD* = 0.30). The pause frequency was 25.6 pauses per 100 syllables (*SD* = 5.7); in effect, these participants paused after every four syllables, and there was some variation among participants. The mean syllable duration was 0.29 s (*SD* = 0.03), which suggests that these participants articulated syllables at a reasonable rate with relatively little variation among participants.

**Table 4:** Descriptive statistics and bootstrapped BCa 95% confidence intervals of fluency measures

	*n*	Mean	*SD*	Range	BCa 95% CI for mean
Mean pause length (seconds)	30	1.19	0.30	0.64–2.11	1.10, 1.28
Pauses per 100 syllables	30	25.6	5.7	13.9–36.9	23.6, 27.6
Mean syllable duration (seconds)	30	0.29	0.03	0.22–0.37	0.28, 0.30

### Role of lexical knowledge and processing in utterance fluency

We investigated whether the L2 lexical knowledge and processing scores predicted the L2 speakers’ utterance fluency. The regression model for mean pause length was significant (*F*(1, 21) = 5.17, *p* = .03). The model explained 16% of the variance in mean pause lengths (adjusted R^2^), which is a small effect size. As reported in Table 5, the model contained one significant predictor, lexical meaning score. No other predictors reached the criterion for inclusion in the model. This analysis showed that greater lexical meaning knowledge was associated with shorter pauses during the unrelated classroom monologue.

**Table 5:** Regression model for mean pause length

	*B*	*SE B*	*β*
Constant	1.51	0.16	
Lexical meaning score	–**0.02**	**0.01**	− 0.44*
	*R*^*2*^ = 0.20, *Adjusted R*^*2*^ = 0.16		

Notes: * *p* < .05, ** *p* < .01; lexis recall, retrieval time, articulation preparation time and articulation duration did not reach the criterion for inclusion in the model. *n* = 22

The regression model for the number of pauses was significant (*F*(1, 21) = 9.43, *p* = .01). The model explained 28% of the variance in the number of pauses (adjusted R^2^), which is a medium effect size. The model (shown in Table 6) contained one significant predictor, articulation preparation time, with no other predictor reaching the criterion for inclusion in the model. This analysis showed that shorter articulation preparation time, i.e. faster lexical processing, was associated with fewer pauses during the monologue.

**Table 6:** Regression model for number of pauses

	*B*	*SE B*	*β*
Constant	16.32	3.40	
Articulation preparation time	**11.87**	**3.87**	0.56**
	*R*^*2*^ = 0.31, *Adjusted R*^*2*^ = 0.28		

Note: * *p* < .05, ** *p* < .01; lexis recall, lexis meaning, retrieval time, and articulation duration did not reach the criterion for inclusion in the model. *n* = 22

The regression model for syllable duration was also significant (*F*(1, 22) = 4.54, *p* = .04) and explained 13% of the variance in syllable duration (adjusted R^2^), a small effect size. The model contained one significant predictor, lexical retrieval time, as reported in Table 7; no other predictor reached the criterion for inclusion in the model. This analysis showed that the ability to retrieve words faster was associated with faster articulated syllables during the monologue.

**Table 7:** Regression model for syllable duration

	*B*	*SE B*	*β*
Constant	0.23	0.03	
Lexical retrieval	**0.03**	**0.02**	0.41*
	*R*^*2*^ = 0.17, *Adjusted R*^*2*^ = 0.13		

Note: * *p* < .05, ** *p* < .01; lexis recall, lexis meaning, articulation preparation time and articulation duration did not reach the criterion for inclusion in the model. *n* = 23

In summary, the regression analyses showed that lexical meaning, a knowledge measure, best explained variance in mean pause length, whereas articulation preparation time and lexical retrieval time, both processing measures, best explained variance in the number of pauses and syllable duration, respectively. Neither lexis recall scores nor articulation duration was found to explain variance in any of the utterance fluency measures for these L2 speakers.

## Discussion

This exploratory study found that a measure of L2 lexical knowledge correlated with an L2 lexical processing measure and that another measure of lexical knowledge and two processing measures explained variance in utterance fluency in the speech of high-intermediate learners of English from various language backgrounds. These findings are discussed below.

### L2 lexical knowledge and processing

Importantly, we assessed the speakers’ size of lexical knowledge with an oral productive recall task, which better approximates the process used in speech (compared with written tasks). Likewise, the lexical meaning test included productive sentential knowledge, which more closely reflects how speakers use lexical knowledge in communication, putting words in a meaningful context. Although Stewart et al. (2012) questioned whether sentential knowledge was distinct from definitional knowledge, the current study found that these learners of English differed in their ability to produce semantically appropriate sentences, even after correctly identifying a word’s meaning. This difference is likely because the current study assessed recognition of the word’s definition (rather than recall), which is more distinct from productive sentential knowledge.

Turning to the analysis, lexis recall correlated with lexical retrieval speed. This finding shows that, for these high-intermediate L2 learners of English, the ability to recall more words was generally associated with the ability to retrieve those words faster, even though they had a larger store of English lexical items. The relationship between greater lexis recall and faster lexical retrieval for these English language learners was somewhat surprising given the mix of language backgrounds, which reduces the likelihood of cognates that facilitate lexical retrieval time. This result extends Llompart’s (2021) finding with advanced L1 German/L2 English learners both to intermediate learners and to learners from various language backgrounds. The finding may suggest that lexical knowledge and lexical retrieval processing speed may develop simultaneously, at least for single-word lexical items.

The correlation between a lexical knowledge (i.e., lexical recall) and a lexical processing measure (i.e., lexical retrieval speed) is contrary to Suzuki and Kormos’s (2023) two-factor model which separates measures of linguistic resources (e.g., lexical knowledge) from measures of lexical processing (e.g., lexical retrieval speed). The research methodology used by Suzuki and Kormos differed considerably from the current study, in that they included only Japanese learners of English, used a written vocabulary test, and included multiple measures of linguistic knowledge and linguistic processing, rather than a direct comparison of L2 lexical knowledge and processing measures.

With the exceptions of the correlation between lexis recall and lexical retrieval speed and the inherent relationship between lexical retrieval and articulation preparation, there was little evidence for correlations among the other chosen measures of L2 lexical knowledge and lexical processing, as shown by the confidence intervals ranging between negative and weakly positive. Notably, the measures of lexical knowledge did not seem to correlate, which is contrary to findings from previous studies (e.g., Koizumi and In’nami 2013; González-Fernández and Schmitt 2020); this contrast may be explained by differences in testing modality (spoken vs. written) and/or test type (e.g. picture-naming vs. blank filling). Lexical retrieval speed was not associated with articulation duration, echoing the findings by Kahng (2020) with Chinese L1 learners of English. Results showing the independence of these measures (especially the articulation measures) across studies, including the current study with learners from various language backgrounds, suggest that they reflect different aspects of cognitive processing. In terms of Levelt’s (1999) model, our results suggest that the lexical knowledge store and the phonological/phonetic system may generally develop at different rates in high-intermediate second language speakers, which would then be expected to differently affect utterance fluency.

### Role of lexical knowledge and lexical processing on utterance fluency

We then investigated possible roles of L2 lexical knowledge and processing on L2 utterance fluency. We found that knowledge of lexical meaning explained variance in pause length in the monologues, which suggests that having depth of lexical knowledge can help speakers construct utterances more quickly, shortening pauses. Our finding contrasts with Kahng (2020), but Kahng’s vocabulary knowledge measure did not include a measure of meaning (only the identification of words from non-words). The lexical meaning test in the current study provided a more detailed picture of lexical knowledge because it was able to capture both word recognition and productive sentential knowledge. Higher lexical meaning scores (driven by sentential knowledge) were associated with shorter pauses in the unrelated speaking task. Given the combination of results, productive sentential knowledge, rather than simply definitional knowledge of individual lexical items, seems to be connected to utterance fluency.

Articulation preparation time explained variance in the number of pauses per 100 syllables: faster articulation preparation time was associated with fewer pauses during the monologues. This result aligns with Clenton et al.’s (2020) finding, but that study used a higher pause threshold (350 ms) than the current study (200 ms), which means the association between articulation preparation time and number of pauses remains even when shorter pauses are included. Fewer pauses per 100 syllables means longer stretches of language. This finding of the role of articulation preparation suggests that the fluency vulnerability was not in the mental lexicon or lexical retrieval per se but in encoding (which is lexically driven in the Levelt model; see also Segalowitz 2010). The current finding with high-intermediate English learners from various L1 backgrounds is compatible with the conclusion drawn by Broos et al. (2021) about advanced Dutch L1 English L2 bilinguals.

Variation in syllable duration during the monologue was explained by lexical retrieval time, which is in line with Kahng’s (2020) findings. Accordingly, syllable duration during speech may reflect factors of cognitive processing other than just articulation. Given Duez’s (1982) description of syllable lengthening as a type of hesitation, it is possible that at least some speakers employ a strategy of lengthening syllables to avoid pausing for lexical retrieval in the monologues. This possibility was also mentioned in De Jong et al. (2012).

We would like to briefly discuss two additional findings. First, we did not find evidence of a relationship between lexis size and any measure of utterance fluency, contrary to previous research (e.g., Clenton et al. 2020; Kahng 2020; Koizumi and In’nami 2013). This contrasting finding might reflect the differing cognitive demands between receptive skills (e.g., reading) and productive skills or possibly the relatively limited variation among the high-intermediate learners in the current study. More importantly, lexis size is likely less relevant in an open-ended monologue, where the speaker has a choice about which words to use. Second, although articulation duration and syllable duration both reflect articulation speed, articulation duration was not a significant predictor of syllable duration in the monologues, which may be due to the relatively small amount of variation among participants for this measure, despite the participants having different language backgrounds. The standard deviation was only 11% relative to the mean for syllable duration, as compared to 25% and 22% of the mean for pause length and number of pauses, respectively. Given that Clenton et al. (2020) found no relationship between articulation duration and syllable duration in the speech of pre-intermediate learners, the findings across these studies may suggest that articulation skills are automatized early in proficiency and, therefore, have little effect on utterance fluency beyond beginner-level learners.

## Conclusion

This exploratory study first investigated L2 lexical processing and lexical knowledge. Lexical recall scores correlated with lexical retrieval speed, which suggests that the ability to recall more words does not slow lexical retrieval. The study then explored whether variance in L2 utterance fluency could be predicted with measures of lexical knowledge and lexical processing, using methodology which more closely mirrored the cognitive processes needed for speech: a form recall task in the oral modality and productive sentential knowledge. Our findings support the theoretical expectations that L2 lexical knowledge, processing, and articulation skills play distinct, significant roles in L2 utterance fluency. Specifically, lexical meaning scores predicted pause length, articulation preparation time predicted number of pauses, and lexical retrieval speed predicted syllable duration during a monologic speech. The results are consistent with the conclusion that articulation skills were already automatized for these L2 learners and with the expectation that the ability to recall specific lexical items is less relevant in productive tasks, such as topic-based speeches. For these learners, L2 utterance fluency appears to rely more on the depth of lexical knowledge, particularly sentential knowledge, as well as the lexical processing skills reflected in lexical retrieval speed and articulation preparation time. Although the findings reflect a single proficiency level (high-intermediate learners), our findings expand upon research with less proficient (e.g., Clenton et al. 2020) and more proficient learners (e.g., Broos et al. 2021; Llompart 2021).

When interpreting the results of this study, some methodological points should be acknowledged. First, with the goal of researching a population of instructed English language learners, our findings are limited to and by the sample size in the IEP. While the inclusion of English language learners from various L1 backgrounds expands generalizability, it adds variance. Overall, the explanatory power of these models is reasonable (see Plonsky and Ghanbar 2018), particularly considering the goal of predicting L2 utterance fluency of speakers from different language backgrounds using only lexical knowledge and processing measures. However, non-significant relationships might be due to the lack of statistical power from the relatively small number of participants. At the same time, the significant findings, despite a limited sample size, suggest that the relationships reported here are durable. Additionally, given that many of the previous studies were limited to learners of English from a single language background (e.g., Broos et al. 2021; Clenton et al. 2020; Enayat and Derakhshan 2021; Gao and Sun 2025; Kahng 2020; Koizumi and In’nami 2013; Takizawa 2024), our study, arguably, reflects a broader population of language learners.

Secondly, our findings are limited to single vocabulary words; research on phrasal vocabulary knowledge (e.g., Kahng 2020; Takizawa and Suzuki 2026) and collocational knowledge (e.g., Koizumi and In’nami 2013; Naismith and Juffs 2025; Takizawa 2024; Uchihara et al. 2022) can offer additional insight into how lexical knowledge of longer lexical constructions affects L2 fluency. Our results may also have been influenced by our choice to sample all lexis from general frequency lists in an attempt to capture general lexis knowledge (cf. Stoeckel et al. 2021). Utterance fluency, however, is likely affected mostly by lexical problems with very specific words, as De Jong and Vercellotti (2016) showed for the word *turtle* in their data and as Hilton (2008) showed for *crane*, and such lexical problems may be amplified when learners have different L1s. In other words, lexical knowledge and processing may be highly individual and contextual. Future studies could further explore topic-relevant lexical knowledge and processing to obtain a more detailed picture of the relationship between L2 lexical knowledge and processing and L2 utterance fluency.

Thirdly, L2 utterance fluency was measured during a topic-prompt monologic speaking task, and so the results describing the roles of L2 lexical knowledge and processing on L2 utterance fluency may be limited to this particular speaking task. Given the possibility of other factors that affect L2 utterance fluency in elicited speech (e.g., Gao and Sun 2025; Uchihara and Clenton 2022; Yan et al. 2025), future research should investigate whether the same relationships are found with other speaking tasks (e.g., Gao and Sun 2025) and other learning contexts.

Expanding on previous research, this study confirms a relationship between lexical knowledge and lexical retrieval speed of L2 speakers of English across L1 backgrounds, and it affirms the importance (and feasibility) of assessing lexical knowledge with methodology that more closely reflects the cognitive processes needed for speech. The results showed that L2 lexical knowledge and lexical processing are both related to distinct aspects of L2 utterance fluency, which supports previous conclusions that speaking theories should include both L2 lexical knowledge and processing. In terms of Levelt’s model, our results suggest that the lexical knowledge store (particularly sentential lexical knowledge) as well as lexical retrieval speed and the phonological/phonetic system (i.e., articulation preparation) clearly and differentially influence utterance fluency for high-intermediate L2 English speakers from various L1 backgrounds.

## Data Availability

The raw data, which include personally identifying information, are not available due to the scope of the consent form. The processed data sets are, however, available from the first author upon reasonable request.

## Footnotes

1 While repair has also been identified as an aspect of fluency, repair behavior appears to reflect the learner’s attention to accuracy rather than an aspect of fluency (Ellis and Barkhuizen 2005; Gilabert 2007) and was not considered in this study.

2 Although collocational knowledge can distinguish learners across proficiency levels (Naismith and Juffs 2025), this study included only one proficiency level so this measure may be less relevant. Additionally, collocations are less frequent than single-word lemma (Naismith 2022), but our study focused on high frequency lexical items.

3 Word frequency was controlled across conditions. The mean word frequency in the COCA corpus was 33,653 (*SD* = 35,863, minimum = 7016, maximum = 145,293) for the immediate naming condition, and 35,543 (*SD* = 41,344, min. = 7,222, max. = 147,379) in the delayed naming condition.

4 A two-tailed independent-samples *t*-test confirmed that the mean length of correct answers (3.9 words, *SD* = 1.4) did not differ from the mean length of the distractors across all items (3.8 words, *SD* = 1.4); *t*(52) = 0.28, *p* = .79.

5 This sentential knowledge task could have been recorded orally, but spoken sentences require more cognitive resources and confound articulation encoding with sentential knowledge. An oral modality has not been validated.
